# Limiar de Desfibrilação em Pacientes com Cardiopatia Chagásica Crônica: Há Benefícios ou Não Vale o Risco?

**DOI:** 10.36660/abc.20220790

**Published:** 2022-11-23

**Authors:** Ricardo Alkmim Teixeira

**Affiliations:** 1 Hospital Renascentista Pouso Alegre MG Brasil Hospital Renascentista, Pouso Alegre, MG – Brasil; 2 Universidade do Vale do Sapucaí Pouso Alegre MG Brasil Universidade do Vale do Sapucaí (UNIVÁS), Pouso Alegre, MG – Brasil

**Keywords:** Doença de Chagas, Cardiomiopatia Chagásica, Taquicardia Ventricular, Desfibriladores, Eletrochoque

O cardioversor-desfibrilador implantável (CDI) é a melhor opção terapêutica para prevenir a morte súbita cardíaca para vários grupos de pacientes de alto risco. Basicamente, o CDI pode reconhecer e interromper a taquicardia ventricular (TV) e a fibrilação ventricular (FV) por meio de estimulação anti-taquicardia (ATP) ou terapia de choque. O limiar de desfibrilação (LDF) é a energia de choque mínima necessária para interromper a FV.

Historicamente, o teste da função do CDI via indução e término da FV era considerado obrigatório para garantir que o CDI pudesse detectar e interromper adequadamente o evento arrítmico.^1^

Várias séries identificaram fatores de risco associados a necessidade de alta energia de desfibrilação que podem afetar o desempenho do CDI: baixa fração de ejeção do ventrículo esquerdo (FEVE), posição do eletrodo em região septal, polaridade do choque catódico, idade avançada, presença de insuficiência cardíaca congestiva, classe funcional mais elevada (NYHA), uso de amiodarona e outros fármacos antiarrítmicos.^2-4^ Por isso, o LDF fazia parte do protocolo de implante em ensaios clínicos clássicos de CDI e também foi amplamente incorporado à prática clínica.

No entanto, a indução de uma arritmia potencialmente fatal para determinar o LDF pode não ser isenta de riscos, afetando a morbidade e mortalidade. As complicações podem estar relacionadas à própria indução da FV e sua duração, aos efeitos da sedação profunda necessária para a realização do teste e aos efeitos adversos de choques adicionais necessários. Além disso, algumas situações clínicas são contraindicações absolutas ou relativas ao LDF, como estenose aórtica grave, doença arterial coronariana crítica, choque cardiogênico e trombo intracardíaco.^5^

Neste contexto histórico e considerando o aprimoramento da tecnologia [a capacidade de detecção provou ser confiável, e a energia necessária para interromper a FV geralmente é baixa (< 15 J)], a relação entre o desempenho e o sucesso do LDF na morbidade e mortalidade a curto e longo prazo foi questionada.^6^

Os dados do SCD-HeFT não mostraram correlação entre o LDF e a mortalidade a longo prazo.^7^ Em duas séries de pacientes submetidos a implante de CDI associado a terapia de ressincronização cardíaca, o LDF também não foi associado a aumento de mortalidade.^8,9^ O impacto do LDF na mortalidade em pacientes submetidos a troca de gerador de CDI ou upgrade de sistema foi avaliado no Registro REPLACE, não havendo associação entre LDF e mortalidade subsequente de 6 meses.^10^ O estudo MODALITY não demonstrou diferença na interrupção da arritmia ventricular entre elerodos de mola única ou dupla (o aumento do risco de falha de choque foi associado à polaridade catódica da mola do ventrículo direito).^2^ No estudo SIMPLE, os investigadores randomizaram 2.500 pacientes submetidos a implante inicial de CDI do lado esquerdo para indicação de prevenção primária ou secundária com ou sem realização do LDF. Não houve diferença estatisticamente significativa no desfecho secundário de mortalidade total entre os grupos (3,0 vs. 2,2%, p = 0,17).^11^ O estudo NORDIC ICD foi projetado para determinar se não realizar LDF foi não-inferior a realizar o LDF para o desfecho primário de eficácia do primeiro choque em encerrar todos os episódios verdadeiros de TV ou FV durante o acompanhamento e, como no estudo SIMPLE, a mortalidade total foi o endpoint secundário pré-definido. Neste estudo, 1.077 pacientes foram randomizados para implantes de CDI do lado esquerdo com ou sem LDF. A mortalidade total não diferiu entre os dois grupos.^12^ Duas metanálises subsequentes demonstraram achados de mortalidade semelhante.^13,14^ Assim, a realização do LDF no momento do implante do CDI não afeta a mortalidade total subsequente.

Existem situações específicas em que o LDF continua sendo uma consideração clínica razoável. Estas incluem implantes do lado direito, mal funcionamento de eletrodos, doenças cardiovasculares hereditárias e congênitas e CDI subcutâneo. Também é importante notar que os estudos que avaliaram o impacto do LDF não incluíram pacientes com cardiomiopatia chagásica crônica (CCC).

Campos et al.^15^ investigaram o uso de LDF em pacientes com CCC, com foco em óbitos relacionados ao CDI e eventos arrítmicos e tratamento durante o seguimento de longo prazo. Os autores avaliaram retrospectivamente 133 pacientes que receberam CDI principalmente para prevenção secundária. A idade média dos pacientes foi de 61 (DP, 13), e 72% eram homens. A FEVE inicial foi de 40 (DP, 15%), e o escore de Rassi médio foi de 10 (DP, 4). Nenhuma morte ocorreu durante o LDF, e nenhuma falha do CDI foi documentada. Houve relação entre escore de Rassi mais elevado e LDF mais alto (ANOVA = 0,007). O tempo médio para o primeiro choque foi de 474 (DP, 628) dias, embora o choque tenha sido necessário apenas para 28 (35%) pacientes com TV, pois a maioria dos casos se resolveu espontaneamente ou por ATP. Após um seguimento clínico médio de 1.728 (DP, 1.189) dias, ocorreram 43 óbitos, principalmente relacionados à insuficiência cardíaca progressiva e sepse.

A doença de Chagas é frequentemente negligenciada em países onde a doença é endêmica devido às inúmeras limitações para o desenvolvimento de pesquisas robustas que possam mudar o padrão de tratamento para melhor. Todas as novas informações devem ser valorizadas e divulgadas, pois países como o Brasil devem ainda lidar com esses pacientes por muito tempo.
