# Supplementary material for: Limiar de Desfibrilação em Pacientes com Cardiopatia Chagásica Crônica: Há Benefícios ou Não Vale o Risco?
Source: Arq Bras Cardiol. 2022 Nov 23;119(6):929–30. [Article in Portuguese] doi: 10.36660/abc.20220790 (PMC9814797; doi:10.36660/abc.20220790)
Supplement: Translation

# Translation: Defibrillation Threshold in Patients with Chronic Chagas’ Heart Disease: Are There Benefits or not Worth the Risk?

**Authors:** Ricardo Alkmim Teixeira

**Affiliations:** 1 Pouso Alegre MG Brazil Hospital Renascentista, Pouso Alegre, MG – Brazil; 2 Pouso Alegre MG Brasil Universidade do Vale do Sapucaí (UNIVÁS), Pouso Alegre, MG – Brazil

**Keywords:** Chagas Disease, Chagas Cardiomyopathy, Ventricular Tachycardia, Defibrillators, Electroshock

The implantable cardioverter-defibrillator (ICD) is the best therapeutic option to prevent sudden cardiac death for several high-risk groups of patients. Basically, the ICD can recognize and stop ventricular tachycardia (VT) and ventricular fibrillation (VF) by triggering anti-tachycardia pacing (ATP) or shock therapy. The defibrillation threshold (DT) is the minimum shock energy required to terminate VF.

Historically, testing the ICD function via induction and termination of VF was considered mandatory to ensure that the ICD could properly detect and stop the arrhythmic event.^1^

Multiple series have identified clinical risk factors for elevated defibrillation energy requirements that could affect ICD performance: low left ventricle ejection fraction (LVEF), septal lead placement, cathodal shock polarity, older age, presence of congestive heart failure, higher NYHA functional class and usage of amiodarone and other antiarrhythmic drugs.^2-4^ Because of that, DT was included as part of the implant protocol in the classic ICD trials and has also been widely incorporated into clinical practice.

However, the induction of a potentially fatal arrhythmia to determine the DT may not be risk-free, affecting morbidity and mortality. Complications can be related to the VF induction itself and its duration, to effects from deep sedation needed to perform the test and to adverse effects of additional necessary shocks. Furthermore, some clinical situations are either absolute or relative contraindications to DT, such as severe aortic stenosis, critical coronary artery disease, cardiogenic shock, and intracardiac thrombus.^5^

In this historical context and considering the technology improvement [the detection capability proved to be reliable, and the energy requirements to terminate VF is generally low (< 15 J)], the relationship between the performance and the success of DT to short- and long-term morbidity and mortality was questioned.^6^

Data from SCD-HeFT showed no correlation between DT and long-term mortality.^7^ In two separate series of patients undergoing initial cardiac resynchronization therapy ICD implantation, DT was also not associated with an increase in mortality.^8,9^ The impact of DT on subsequent mortality in patients undergoing ICD generator replacement or upgrade was evaluated in the REPLACE Registry, and there was no association between DT and subsequent mortality at 6 months.^10^ The MODALITY trial demonstrated no difference in ventricular arrhythmia termination between single- and dual-coil leads (increased risk of shock failure was associated with right ventricular coil cathodal polarity).^2^ In the SIMPLE trial, investigators randomized 2500 patients undergoing an initial implant of a left-sided ICD for either primary or secondary prevention indication to DT or no DT. There was no statistically significant difference in the secondary endpoint of total mortality between the groups (3.0 vs. 2.2%, p = 0.17).^11^ The NORDIC ICD trial was designed to determine if no DT was non-inferior to DT for the primary endpoint of first shock efficacy in terminating all true episodes of VT or VF during follow-up, and as in the SIMPLE trial, total mortality was a pre-defined secondary endpoint. In this trial, 1077 patients were randomized to left-sided ICD implants with or without DT. Total mortality did not differ between the two groups.^12^ Two subsequent meta-analyses demonstrated similar mortality findings.^13,14^ Thus, the performance of DT at the time of ICD implant does not affect subsequent total mortality.

There are specific situations where DT remains a reasonable clinical consideration. These include right-sided implants, lead advisories and malfunction, inherited and congenital cardiovascular disease, and subcutaneous ICD. It is also important to note that those studies evaluating the impact of DT did not include chronic Chagas cardiomyopathy (CCC) patients.

Campos et al.^15^ investigated the use of DT in CCC patients, focusing on deaths related to ICD and arrhythmic events and treatment during long-term follow-up. The authors retrospectively evaluated 133 patients who received an ICD mainly for secondary prevention. The mean patient age was 61 (SD, 13), and 72% were men. The baseline LVEF was 40 (SD, 15%), and the mean Rassi score was 10 (SD, 4). No deaths occurred during DT, and no ICD failures were documented. There was a relationship between higher baseline Rassi scores and higher DT scores (ANOVA = 0.007). The mean time to first shock was 474 (SD, 628) days, although shock was only necessary for 28 (35%) patients with VT since most cases resolved spontaneously or through ATP. After a mean clinical follow-up of 1728 (SD, 1189) days, 43 deaths occurred, mainly related to progressive heart failure and sepsis. Based on that results, the authors concluded that a routine DT might not be necessary for CCC patients who receive an ICD for secondary prevention.

Chagas disease is often neglected in countries where the disease is endemic due to the numerous limitations for developing robust research that can change the standard of treatment for the better. All new information must be valued and disseminated since countries like Brazil must deal with these patients for a long time.
